# DIFFERENT TYPES OF INTERNET USE AND SOCIAL ADAPTATION AMONG OLDER ADULTS

**DOI:** 10.1093/geroni/igad104.2175

**Published:** 2023-12-21

## Abstract

Adaptation is pivotal in achieving successful aging. However, the surge in Internet use and social distancing measures during COVID-19 exacerbate the existing adaptation difficulties of older adults with insufficient Internet exposure. Despite this, little attention has been paid to older adults’ Internet use and related social adaptation. This study aimed to explore how older adults’ different types of Internet use influence their social adaptation and determine how to increase their Internet use from the user’s perspective to promote social adaptation. We recruited 388 adults aged 60 to 83 and measured their frequency of four principal types of Internet use: information-seeking, social interaction, instrumental use, and leisure & entertainment. Structural equation modeling was employed to compare effects of different types of Internet use on older adults’ social adaptation, as well as examine the predictive roles of Internet control belief and involvement. These results revealed instrumental use and leisure & entertainment were significantly and positively associated with the social adaptation of older adults among the four types of Internet use, whereas information-seeking and social interaction were not. Additionally, Internet control belief and involvement positively predicted all four types of Internet use among older adults and were associated with social adaptation through instrumental use and leisure & entertainment. The findings imply that instrumental use and leisure & entertainment are particularly important for promoting social adaptation among older adults. To increase Internet use and promote social adaptation, interventions should prioritize these types of Internet use and focus on enhancing Internet control belief and involvement.

